# Endpoint-Dependent Biochemical and Histopathological Effects of Orientin in a Short-Term 3-Nitropropionic Acid Mouse Model

**DOI:** 10.3390/biology15191776

**Published:** 2026-10-07

**Authors:** Abdulsamed Kükürt, Volkan Gelen, Lale Başer, Recai Aci, Murat Arı, Hilmi Nuhoğlu, Ayfer Yıldız Uysal, Ahmet Melik Acar, Halil Ayvazoğlu, Yeşim Can Polat, Serpil Dağ, Mahmut Karapehlivan

**Affiliations:** 1 Department of Biochemistry, Faculty of Veterinary Medicine, Kafkas University, 36100 Kars, Türkiye; 2 Department of Physiology, Faculty of Veterinary Medicine, Kafkas University, 36100 Kars, Türkiye; 3 Söke Health Services Vocational School, Aydın Adnan Menderes University, 09100 Aydın, Türkiye; 4 Department of Pathology, Faculty of Veterinary Medicine, Kafkas University, 36100 Kars, Türkiye; hilminuhoglu@gmail.com (H.N.);; 5 Department of Medical Biochemistry, Faculty of Medicine, Kafkas University, 36100 Kars, Türkiye

**Keywords:** orientin, 3-nitropropionic acid, striatum, oxidative stress, MCP-1, histopathology

## Abstract

Objective: The objective of this study was to describe the biochemical and histopathological changes associated with orientin co-administration in a short-term 3-nitropropionic acid (3-NPA) mouse model. Methods: Twenty-four adult female Swiss albino mice were allocated to Control, Orientin, 3-NPA, and 3-NPA + Orientin groups (*n* = 6). Mice received 3-NPA (12.5 mg/kg/day, i.p.) and/or orientin (20 mg/kg/day, i.p.) for seven days. After midsagittal division of the brain, GSH, MDA, SOD, GPx, and CAT were measured in a homogenate prepared from one hemisphere per animal; caspase-3, COX-2, iNOS, and MCP-1 were measured in plasma. The contralateral hemisphere was used for striatal histology and assessment of 8-OHdG, 4-HNE, and GFAP immunoreactivities; right–left allocation was balanced within each group. Results: Factorial analysis identified endpoint-dependent 3-NPA and orientin effects. Relative to 3-NPA alone, co-treatment increased GSH, SOD, and CAT and reduced MDA, caspase-3, and MCP-1. GPx, COX-2, and iNOS did not differ significantly between the 3-NPA and combined-treatment groups. Histopathological injury appeared less extensive with co-treatment. Although 8-OHdG and 4-HNE staining was lower descriptively, the pairwise IRS comparisons between the 3-NPA and combined-treatment groups were not significant; GFAP did not differ among groups. Conclusion: Orientin co-administration was associated with a partial, endpoint-dependent biochemical and histopathological profile. Because behavioral outcomes and direct pathway or target-intervention experiments were not performed, these findings do not establish functional neuroprotection or a specific molecular mechanism.

## Simple Summary

3-Nitropropionic acid disrupts energy production in brain cells and is used to produce short-term brain injury in laboratory animals. We tested whether orientin, a plant-derived antioxidant, altered these changes in mice. Animals received 3-nitropropionic acid, orientin, both substances, or saline for seven days. Orientin co-treatment improved several, but not all, measured outcomes. Some antioxidant measures increased, while selected markers of oxidation, cell death, and inflammation decreased, and tissue injury appeared less extensive. However, other biochemical and tissue measures did not change significantly. The response was therefore partial and depended on the outcome examined. Because the study did not include behavioral testing or experiments targeting specific molecular pathways, it does not establish functional brain protection or explain a specific mechanism.

## 1. Introduction

Disruption of mitochondrial energy metabolism, increased oxidative burden, and altered glia–neuron interactions are common features of processes involving striatal neuronal injury. Reviews of Huntington’s disease and related experimental models indicate that oxidative-damage products, including lipid-peroxidation products and 8-OHdG, should be interpreted together with antioxidant defenses such as GSH [1]. Impairment of the NRF2 and PGC-1α axes has also been linked to mitochondrial dysfunction, neuroinflammation, and redox imbalance [2]. 3-Nitropropionic acid (3-NPA) irreversibly inhibits succinate dehydrogenase and suppresses mitochondrial complex II function, resulting in reduced ATP production, reactive oxygen species accumulation, calcium dysregulation, excitotoxicity, and inflammatory responses. It is therefore used as a toxicological model of striatal susceptibility to metabolic stress [3]. The model does not reproduce mutant huntingtin accumulation or the slowly progressive genetic course of Huntington’s disease; its findings represent selected consequences of mitochondrial toxicity rather than the disease in its entirety.

Striatal selectivity does not arise solely from complex II inhibition; cell-cycle regulators and regional stress responses also influence neuronal vulnerability. Dietrich et al. [4] reported that changes in cyclin D1 levels markedly modulate 3-NPA-associated striatal neurodegeneration. This finding indicates that a process initiated by energy failure expands through cellular stress, apoptotic signaling, and tissue responses. Accordingly, the combined assessment of biochemical oxidation products, endogenous antioxidant defenses, apoptotic markers, and histopathological injury in 3-NPA models is more informative than an approach based on a single endpoint.

Orientin (luteolin-8-C-glucoside) is a water-soluble flavone C-glycoside found in various plants. Recent pharmacological evaluations have highlighted its free-radical-scavenging capacity, support of the Nrf2-related antioxidant response, and ability to limit inflammatory signaling through the NF-κB/MAPK axes [5]. Another recent review summarized experimental evidence supporting the antioxidant, anti-inflammatory, and neuroprotective effects of orientin and emphasized the need to clarify its oral bioavailability, pharmacokinetic profile, and pharmacodynamic profile before clinical development [6]. The hydrophilic structure of orientin may pose an important limitation to its bioavailability and passage across the blood–brain barrier. Moreover, most available evidence is derived from cell-culture and laboratory-animal studies, and dose, route of administration, and model characteristics vary among studies. Therefore, when orientin is evaluated as a neuroprotective candidate, nonresponsive endpoints should be reported alongside favorable changes.

This study aimed to describe the effects of orientin co-administration on selected oxidative-stress, apoptotic, and inflammatory endpoints and on striatal histopathology in a short-term 3-NPA mouse model. GSH, MDA, SOD, GPx, and CAT were measured in homogenates prepared from one hemisphere per animal, and caspase-3, COX-2, iNOS, and MCP-1 were measured in plasma. The contralateral hemisphere was used to evaluate H&E findings and the immunoreactivities of 8-OHdG, 4-HNE, and GFAP. The study was not designed to test Nrf2, NF-κB, MCP-1 causality, or another specific signaling mechanism.

## 2. Materials and Methods

### 2.1. Ethical Approval and Experimental Animals

The study was approved by the Kafkas University Local Ethics Committee for Animal Experiments (decision no. KAÜ-HADYEK/2026 –078). Experimental procedures were conducted in accordance with national legislation and the ARRIVE 2.0 guidelines [7]. Twenty-four adult female Swiss albino mice (2–3 months old, 25–30 g) obtained from the Kafkas University Experimental Animal Application and Research Center were used. Animals were maintained at 22 ± 2 °C and 50–60% relative humidity under a 12 h light/12 h dark cycle, with ad libitum access to standard pellet feed and water. After a one-week acclimatization period, the animals were randomly allocated to four groups (*n* = 6). Sample size was determined on the basis of the local ethics committee restriction permitting a maximum of six animals per group.

### 2.2. Experimental Design

**Figure 1:**
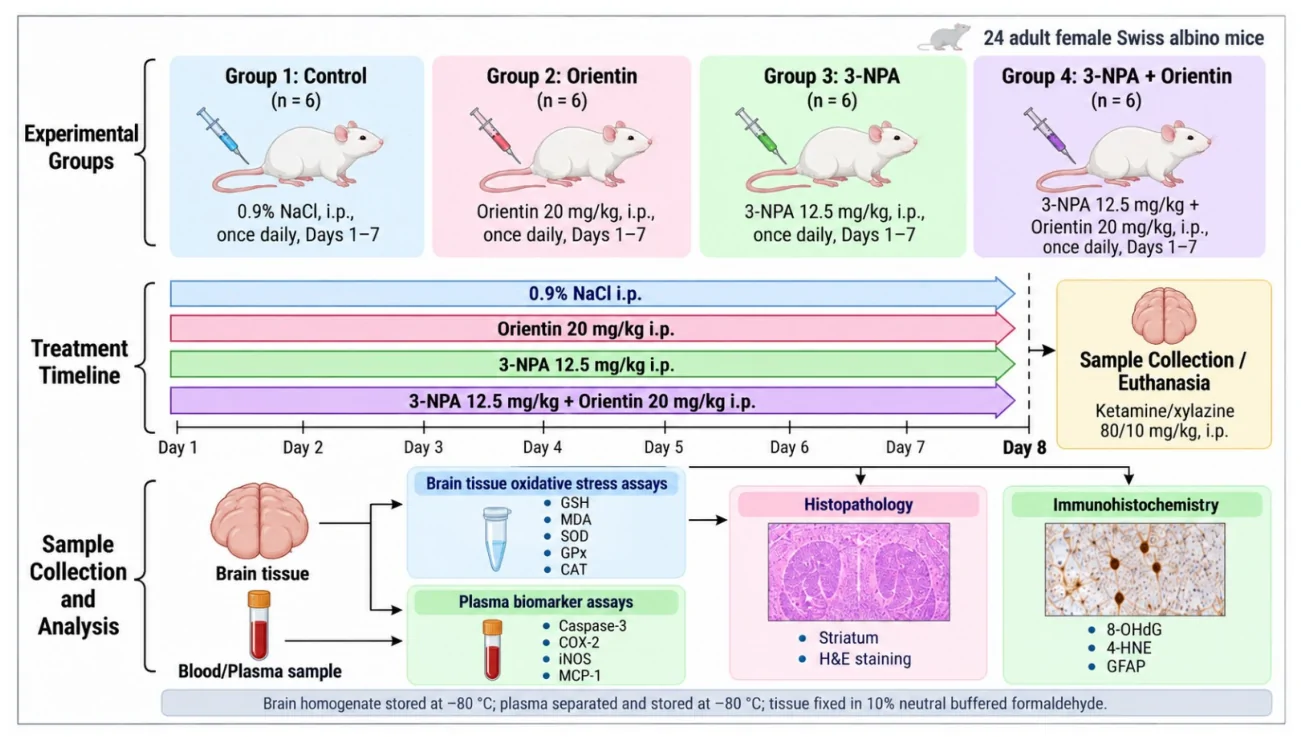
Study design, treatment protocol, and sample-analysis workflow in the experimental model of 3-nitropropionic acid (3-NPA)-induced neurotoxicity. After rapid brain removal, each brain was divided in the midsagittal plane; one hemisphere was used for biochemical assays and the contralateral hemisphere for histopathology and immunohistochemistry, with right–left allocation balanced within each group.

Experimental groups and treatments. The Control group received 0.9% NaCl (i.p.) once daily for seven days; the Orientin group received orientin (20 mg/kg/day, i.p.); the 3-NPA group received 3-NPA (12.5 mg/kg/day, i.p.); and the 3-NPA + Orientin group received 3-NPA (12.5 mg/kg/day, i.p.) and orientin (20 mg/kg/day, i.p.) in combination. Each group comprised six animals (Figure 1).

Dose and protocol sources and sampling. The orientin dose was selected from a study evaluating 20 mg/kg in Swiss albino mice [8], whereas the 3-NPA dose and seven-day administration period were based on Dietrich et al. [4]. At the end of treatment, deep anesthesia was induced with ketamine/xylazine (80/10 mg/kg, i.p.), and the animals were euthanized. Blood was collected for plasma ELISA measurements. Immediately after rapid brain removal, each brain was divided in the midsagittal plane. To balance potential lateralization effects, the right hemisphere was allocated to biochemical analyses and tissue-homogenate preparation for three animals in each group, whereas the left hemisphere was used for the remaining three animals. The hemisphere assigned to biochemical analysis was immediately separated and stored at −80 °C until analysis. The contralateral hemisphere was fixed in 10% neutral-buffered formalin for histopathological and immunohistochemical examination. Coronal sections containing the cerebral cortex and striatum were prepared from the fixed hemispheres; the reported microscopic assessment focused on the striatum. Biochemical measurements, histopathological assessments, and statistical analyses were performed by investigators blinded to group allocation.

### 2.3. Brain-Tissue Homogenization

Frozen hemispheric brain samples were thawed on ice and mechanically homogenized in 0.1 M phosphate buffer (pH 7.4) at a ratio of 1:10 (*w*/*v*). Regional microdissection was not performed within the hemisphere assigned to biochemical analysis. Homogenates were centrifuged at 14,000× *g* for 15 min at 4 °C, and the supernatants were used for GSH, MDA, SOD, GPx, and CAT measurements.

### 2.4. Oxidative-Stress Analyses

All spectrophotometric measurements were performed using a Bio-Tek Gen5 Epoch instrument (BioTek Instruments, Inc., Winooski, VT, USA). MDA was determined using the method of Ohkawa et al. [9], based on measurement of the complex formed with thiobarbituric acid at 532 nm. GSH levels were determined using the method of Beutler et al. [10], in which the yellow product formed by sulfhydryl groups and DTNB is measured at 412 nm. CAT activity was assessed by monitoring the decomposition rate of hydrogen peroxide at 240 nm [11]. GPx activity was determined by monitoring NADPH consumption at 340 nm during the reduction of hydrogen peroxide in the presence of GSH [12]. SOD activity was determined using a method that measures inhibition of the superoxide-mediated reaction at 560 nm [13].

### 2.5. Apoptotic and Inflammatory Markers

Plasma concentrations of MCP-1, caspase-3, COX-2, and iNOS were determined using commercial ELISA kits in accordance with the manufacturer’s protocols (Wayon-Bio Co., Ltd., Shanghai, China; WOB-20248, WOB-20195, WOB-20978, and WOB-21486, respectively).

### 2.6. Histopathological and Immunohistochemical Examination

Formaldehyde-fixed coronal brain tissues containing the cerebral cortex and striatum were embedded in paraffin after routine processing. Four-micrometer sections were stained with H&E. Histopathological and immunohistochemical evaluations were performed in the striatal region; cortical staining is described only where visible in the same coronal sections.

For immunohistochemical examination, 4 µm sections mounted on poly-L-lysine-coated slides were deparaffinized and rehydrated. Endogenous peroxidase activity was blocked with 3% H_2_O_2_ for 20 min. Antigen retrieval was performed in citrate buffer (pH 6.0) by boiling in a 700 W microwave oven for 10 min. After PBS washes, the sections were incubated for 10 min in the protein-blocking solution supplied with the Anti-Polyvalent HRP kit and then incubated overnight at 4 °C with primary antibodies against 4-HNE (K009925P, 1:150), 8-OHdG (bs-1278R, 1:400), or GFAP (AB5804, 1:2000). After application of a biotinylated secondary antibody and streptavidin-peroxidase, the reaction was visualized with AEC chromogen and counterstained with Gill 3 hematoxylin.

Sections were evaluated by two pathologists blinded to group allocation. The proportion of positive cells was scored as 0 (none), 1 (1–25%), 2 (26–50%), or 3 (>50%), and staining intensity was scored as 0 (none), 1 (weak), 2 (moderate), or 3 (strong). The immunoreactive score was calculated by multiplying the positivity and intensity scores (IRS = PS × SI; range, 0–9). Interobserver discrepancies were resolved by joint re-evaluation [14].

### 2.7. Statistical Analysis

The four experimental groups formed a balanced 2 × 2 factorial arrangement defined by 3-NPA exposure (absent/present) and orientin treatment (absent/present; *n* = 6 per cell). For continuous variables satisfying distributional and variance assumptions, two-way ANOVA was used to estimate the 3-NPA main effect, the orientin main effect, and the 3-NPA × orientin interaction. The interaction was evaluated first. When the interaction was significant, interpretation focused on the simple group pattern rather than isolated marginal main effects. Tukey-adjusted pairwise comparisons were then used to localize differences, with Control versus 3-NPA (model induction) and 3-NPA versus 3-NPA + Orientin (orientin effect in the presence of 3-NPA) defined as the biologically primary comparisons. Partial eta-squared (ηp^2^) was reported as the factorial effect size. GPx, which did not meet parametric assumptions, and ordinal IRS data were analyzed using the Kruskal–Wallis test followed by Dunn–Bonferroni pairwise comparisons. Tests were two-sided, and *p* < 0.05 was considered statistically significant. Descriptive statistics are reported as mean ± SD for continuous variables and median with interquartile range for IRS data. Full SS and MS results are provided in Supplementary Tables S1 and S2.

## 3. Results

Interactions were considered before main effects. Table 1 presents the four cell means and SDs, and Table 2 reports the two main effects and interaction for every parametric endpoint with F statistics, degrees of freedom, exact *p* values, and partial eta-squared.

### 3.1. Oxidative-Stress Markers in Brain Tissue

The interaction was examined first. Significant 3-NPA × orientin interactions were identified for GSH (F(1,20) = 10.547, exact *p* = 0.00403, ηp^2^ = 0.345), MDA (F(1,20) = 30.187, exact *p* = 2.23 × 10^−5^, ηp^2^ = 0.601), SOD (F(1,20) = 8.411, exact *p* = 0.00885, ηp^2^ = 0.296), and CAT (F(1,20) = 12.694, exact *p* = 0.00195, ηp^2^ = 0.388). The 3-NPA main effect was significant for all four endpoints. The orientin main effect was significant for MDA, SOD, and CAT, but not for GSH. Complete main-effect and interaction statistics are presented in Table 2. GPx was evaluated separately with the Kruskal–Wallis/Dunn–Bonferroni procedure.

#### 3.1.1. GSH Levels

**Figure 2:**
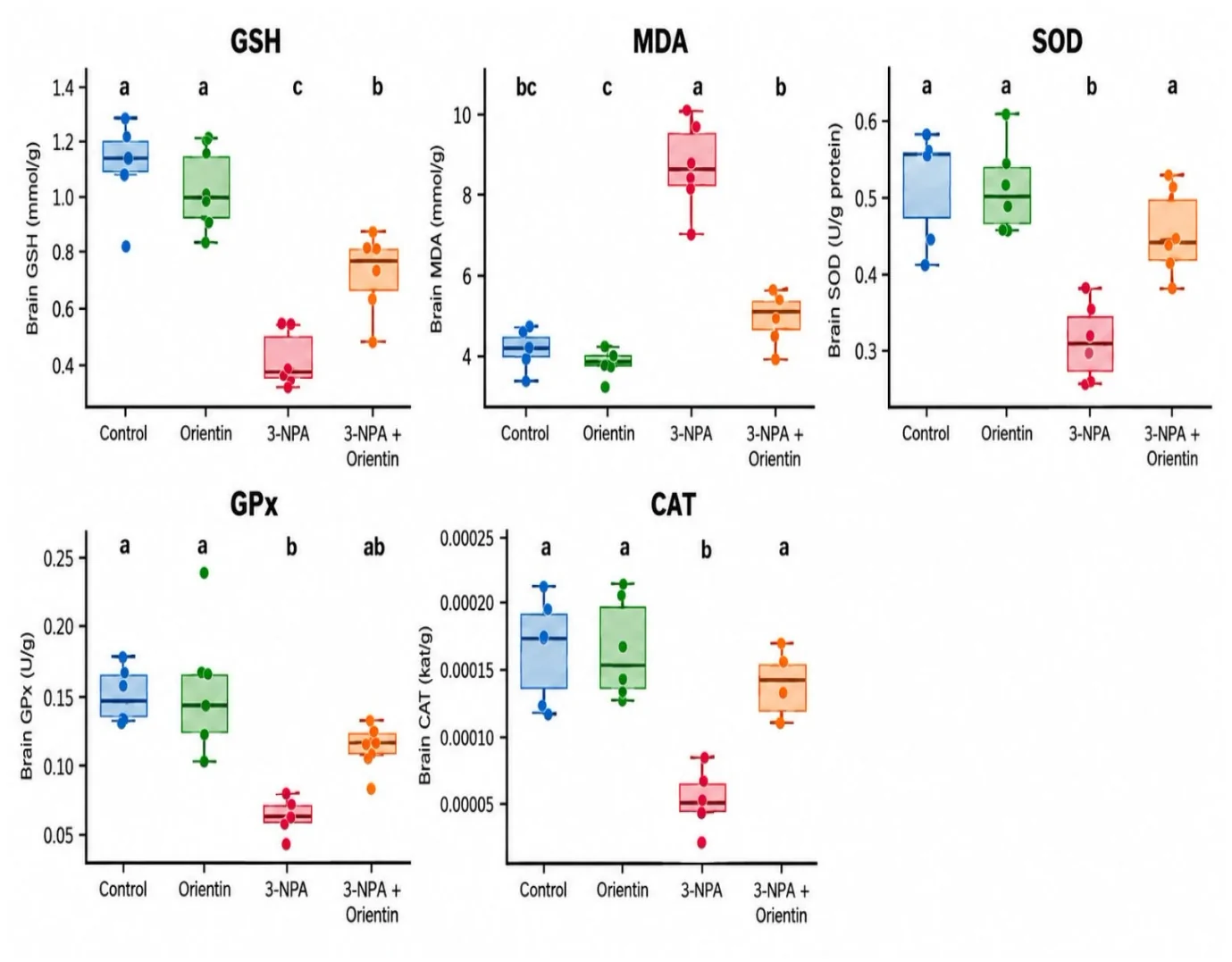
Brain oxidative-stress endpoints by experimental group (*n* = 6/group). Y-axes identify the endpoint and unit; CAT tick labels are presented in decimal notation. Boxes show the median and interquartile range, and dots show individual animals. For GSH, MDA, SOD, and CAT, the 3-NPA × orientin interaction was interpreted before the two main effects, and lowercase letters summarize Tukey-adjusted pairwise comparisons. GPx was analyzed by Kruskal–Wallis followed by Dunn–Bonferroni testing. The compact-letter display denotes multiplicity-adjusted homogeneous subsets rather than an ordinal ranking: identical or overlapping letters indicate an adjusted *p* ≥ 0.05, whereas the absence of a shared letter indicates an adjusted *p* < 0.05. Accordingly, an ab group overlaps statistically with both a- and b-labeled groups but differs from a c-only group; a bc group overlaps with both b- and c-labeled groups but differs from an a-only group. The letters do not indicate the direction or magnitude of the difference, which should be interpreted from the observed values. Compact-letter assignments were as follows: GSH, Control a, Orientin a, 3-NPA c, and 3-NPA + Orientin b; MDA, Control bc, Orientin c, 3-NPA a, and 3-NPA + Orientin b; SOD, Control a, Orientin a, 3-NPA b, and 3-NPA + Orientin a; GPx, Control a, Orientin a, 3-NPA b, and 3-NPA + Orientin ab; and CAT, Control a, Orientin a, 3-NPA b, and 3-NPA + Orientin a. The biologically primary comparisons were Control versus 3-NPA and 3-NPA versus 3-NPA + Orientin. Exact factorial statistics and effect sizes are reported in Table 2 and Supplementary Table S1.

Tissue GSH values are summarized in Table 1 and Figure 2. Consistent with the significant interaction, 3-NPA reduced GSH relative to Control (Tukey-adjusted *p* < 0.001), whereas GSH was higher in the 3-NPA + Orientin group than in the 3-NPA group (*p* = 0.010).

#### 3.1.2. MDA Levels

MDA values are summarized in Table 1 and Figure 2. Consistent with the significant interaction, 3-NPA increased MDA relative to Control (Tukey-adjusted *p* < 0.001), whereas MDA was lower in the 3-NPA + Orientin group than in the 3-NPA group (*p* < 0.001).

#### 3.1.3. SOD Activity

SOD activity is summarized in Table 1 and Figure 2. Consistent with the significant interaction, 3-NPA reduced SOD activity relative to Control (Tukey-adjusted *p* < 0.001), whereas SOD activity was higher in the 3-NPA + Orientin group than in the 3-NPA group (*p* = 0.005).

#### 3.1.4. GPx Activity

GPx activity is summarized in Table 1 and Figure 2. The Kruskal–Wallis test showed an overall group difference (*p* = 0.001). Dunn–Bonferroni testing showed lower GPx activity in the 3-NPA group than in Control (adjusted *p* = 0.001), whereas the 3-NPA versus 3-NPA + Orientin comparison was not significant (adjusted *p* = 0.200).

#### 3.1.5. CAT Activity

CAT activity is summarized in Table 1 and Figure 2. Consistent with the significant interaction, 3-NPA reduced CAT activity relative to Control (Tukey-adjusted *p* < 0.001), whereas CAT activity was higher in the 3-NPA + Orientin group than in the 3-NPA group (*p* < 0.001).

### 3.2. Apoptotic and Inflammatory Markers

The interaction was examined first. A significant 3-NPA × orientin interaction was identified for caspase-3 (F(1,20) = 11.286, exact *p* = 0.00312, ηp^2^ = 0.361), whereas the interactions were not significant for COX-2 (F(1,20) = 2.566, exact *p* = 0.1249, ηp^2^ = 0.114), iNOS (F(1,20) = 0.214, exact *p* = 0.6486, ηp^2^ = 0.011), or MCP-1 (F(1,20) = 0.547, exact *p* = 0.4681, ηp^2^ = 0.027). The 3-NPA main effect was significant for all four plasma endpoints. The orientin main effect was significant for caspase-3 and MCP-1, but not for COX-2 or iNOS. Complete statistics are presented in Table 2.

#### 3.2.1. Caspase-3 Levels

**Figure 3:**
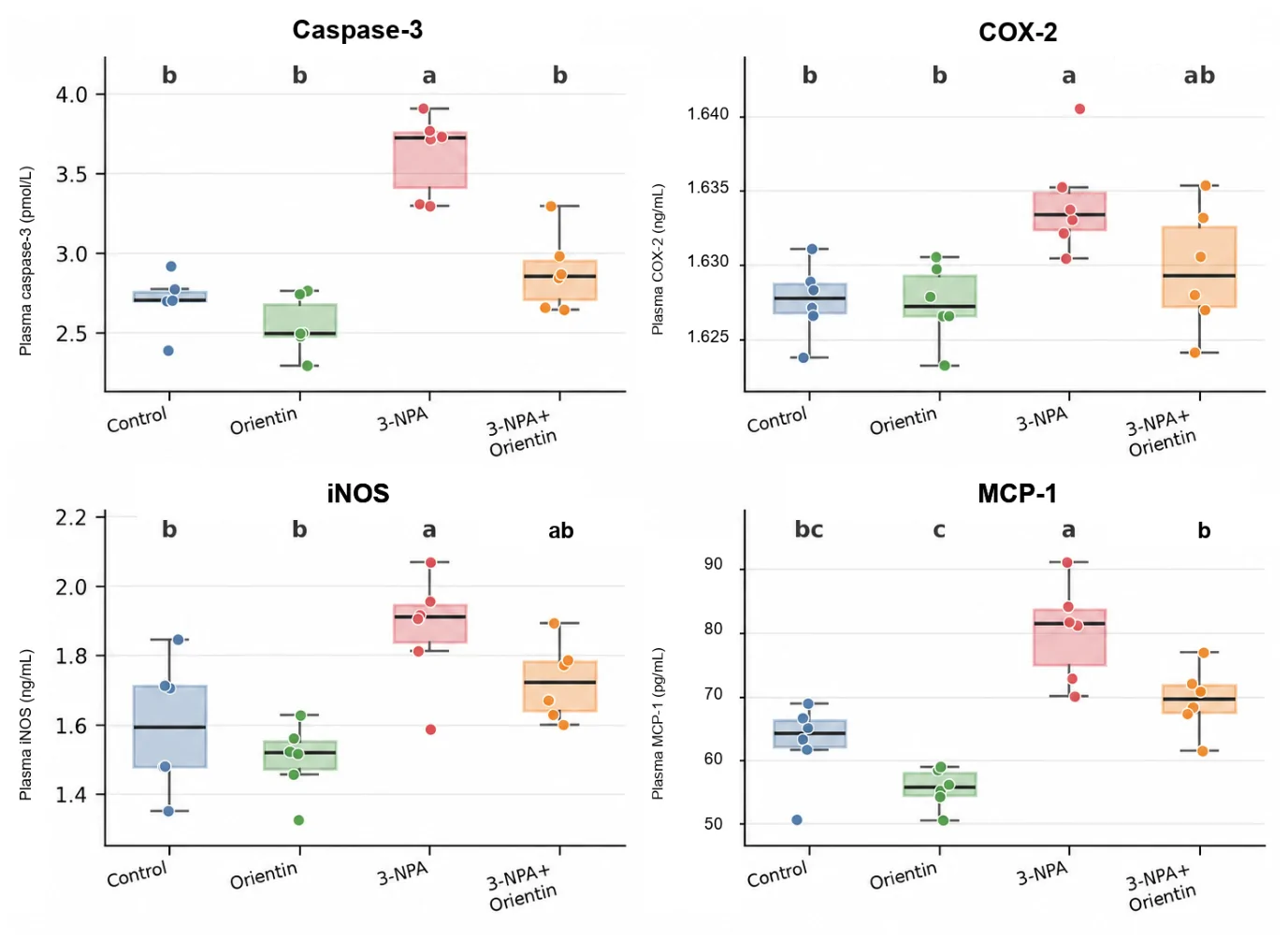
Plasma apoptotic and inflammatory endpoints by experimental group (*n* = 6/group). Y-axes identify the endpoint and unit. Boxes show the median and interquartile range, and dots show individual animals. Two-way ANOVA evaluated the 3-NPA × orientin interaction first, followed by the two main effects. Lowercase letters summarize Tukey-adjusted pairwise comparisons. The compact-letter display denotes multiplicity-adjusted homogeneous subsets rather than an ordinal ranking: identical or overlapping letters indicate an adjusted *p* ≥ 0.05, whereas the absence of a shared letter indicates an adjusted *p* < 0.05. Accordingly, an ab group overlaps statistically with both a- and b-labeled groups but differs from a c-only group; a bc group overlaps with both b- and c-labeled groups but differs from an a-only group. The letters do not indicate the direction or magnitude of the difference, which should be interpreted from the observed values. Compact-letter assignments were as follows: caspase-3, Control b, Orientin b, 3-NPA a, and 3-NPA + Orientin b; COX-2, Control b, Orientin b, 3-NPA a, and 3-NPA + Orientin ab; iNOS, Control b, Orientin b, 3-NPA a, and 3-NPA + Orientin ab; and MCP-1, Control bc, Orientin c, 3-NPA a, and 3-NPA + Orientin b. The biologically primary comparisons were Control versus 3-NPA and 3-NPA versus 3-NPA + Orientin. Exact factorial statistics and effect sizes are reported in Table 2 and Supplementary Table S2.

Plasma caspase-3 values are summarized in Table 1 and Figure 3. Consistent with the significant interaction, 3-NPA increased caspase-3 relative to Control (Tukey-adjusted *p* < 0.001), whereas caspase-3 was lower in the 3-NPA + Orientin group than in the 3-NPA group (*p* < 0.001).

#### 3.2.2. COX-2 Levels

Plasma COX-2 values are summarized in Table 1 and Figure 3. With no significant interaction, the significant 3-NPA main effect (Table 2) was consistent with higher COX-2 in the 3-NPA group than in Control (Tukey-adjusted *p* = 0.012). The biologically primary 3-NPA versus 3-NPA + Orientin comparison was not significant (*p* = 0.112).

#### 3.2.3. iNOS Levels

Plasma iNOS values are summarized in Table 1 and Figure 3. With no significant interaction, the significant 3-NPA main effect (Table 2) was consistent with higher iNOS in the 3-NPA group than in Control (Tukey-adjusted *p* = 0.017). The biologically primary 3-NPA versus 3-NPA + Orientin comparison was not significant (*p* = 0.312).

#### 3.2.4. MCP-1 Levels

Plasma MCP-1 values are summarized in Table 1 and Figure 3. With no significant interaction, both the 3-NPA and orientin main effects were significant (Table 2). MCP-1 was higher in the 3-NPA group than in Control (Tukey-adjusted *p* < 0.001) and was lower in the 3-NPA + Orientin group than in the 3-NPA group (*p* = 0.024).

### 3.3. Histopathological and Immunohistochemical Findings

**Figure 4:**
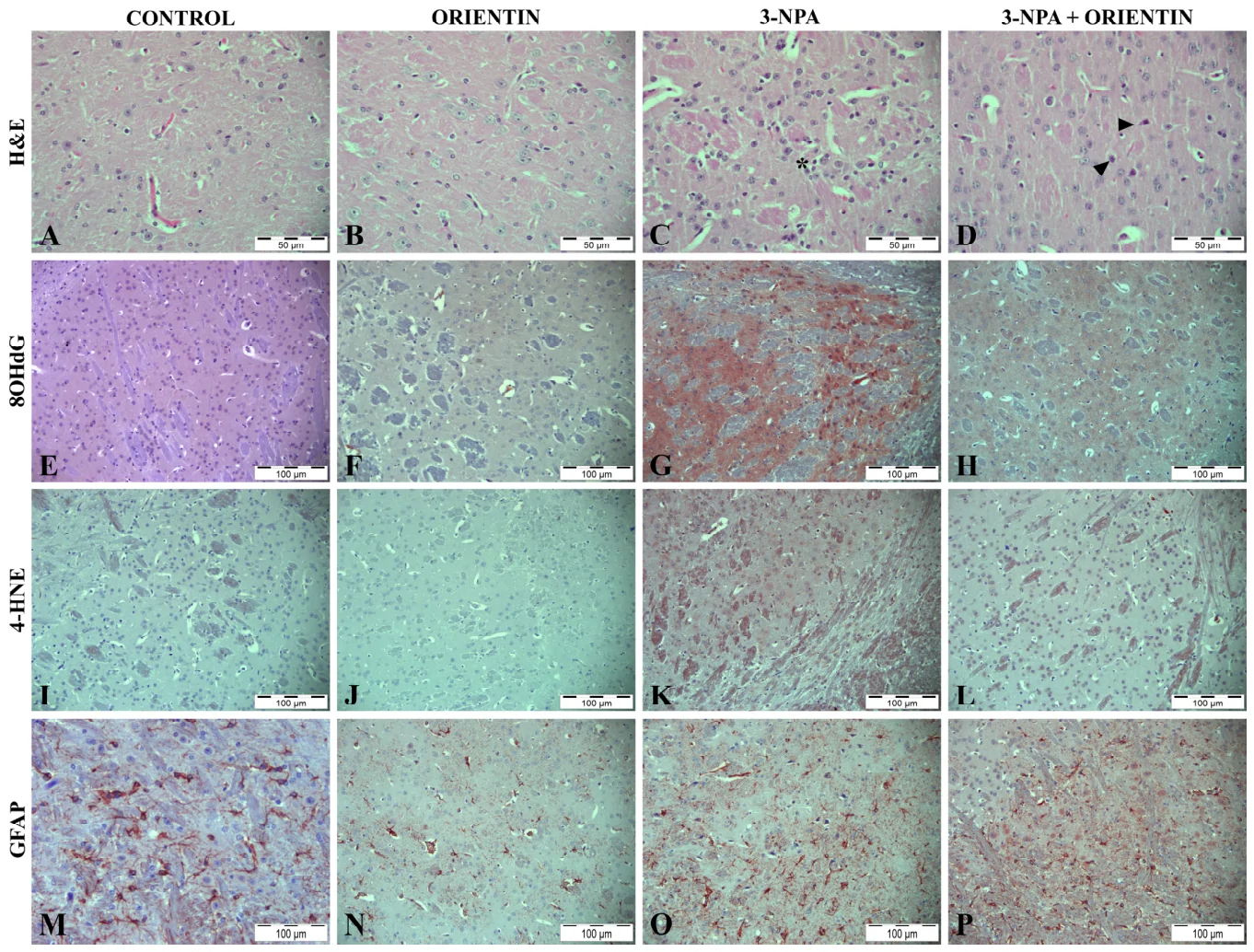
Representative striatal histology and immunohistochemistry. Columns show Control, Orientin, 3-NPA, and 3-NPA + Orientin, respectively. (**A**–**D**) H&E: (**C**) shows gliosis (asterisk), and (**D**) shows residual neuronal necrosis (arrowheads). (**E**–**H**) 8-OHdG: the strongest reaction is visible in (**G**) outside nigrostriatal tract bundles; (**H**) shows the combined-treatment pattern. (**I**–**L**) 4-HNE: (**K**) shows prominent staining in nigrostriatal tract bundles; (**L**) shows the combined-treatment pattern. (**M**–**P**) GFAP. Scale bars: (**A**–**D**) 50 µm; (**E**–**P**) 100 µm. Images are representative; statistical inference is based on IRS analyses described in the Results.

The histopathological architecture of the cerebral cortex and striatum was preserved in the Control and Orientin groups; neurons had prominent central nuclei and regular cytoplasmic borders, and no disruption of neuropil integrity was observed (Figure 4A,B). In the 3-NPA group, neuronal degeneration characterized by cytoplasmic eosinophilia, pyknosis, karyorrhexis, and chromatolysis, together with neuronophagic foci and diffuse/focal gliosis, was observed particularly in the striatal parenchyma (Figure 4C). Although necrotic and chromatolytic neurons were not completely eliminated in the 3-NPA + Orientin group, these changes were less extensive than in the 3-NPA group, and neuronal morphology was better preserved (Figure 4D).

**Figure 5:**
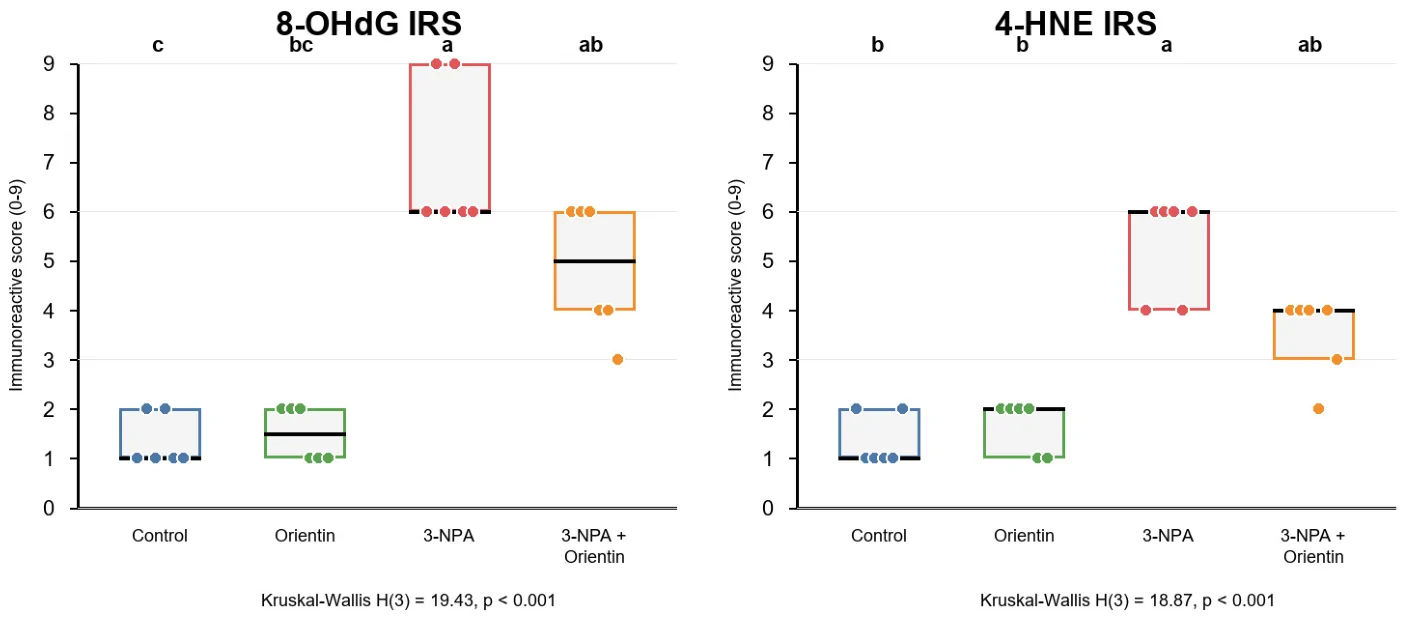
Individual immunoreactive scores (IRSs) for 8-OHdG and 4-HNE in the Control, Orientin, 3-NPA, and 3-NPA + Orientin groups (*n* = 6/group). Boxes show the median and interquartile range, and dots show individual animals. Lowercase letters summarize Dunn–Bonferroni comparisons following a significant Kruskal–Wallis test. The compact-letter display denotes multiplicity-adjusted homogeneous subsets rather than an ordinal ranking: identical or overlapping letters indicate an adjusted *p* ≥ 0.05, whereas the absence of a shared letter indicates an adjusted *p* < 0.05. Accordingly, an ab group overlaps statistically with both a- and b-labeled groups but differs from a c-only group; a bc group overlaps with both b- and c-labeled groups but differs from an a-only group. The letters do not indicate the direction or magnitude of the difference, which should be interpreted from the observed values. Compact-letter assignments were as follows: 8-OHdG, Control c, Orientin bc, 3-NPA a, and 3-NPA + Orientin ab; 4-HNE, Control b, Orientin b, 3-NPA a, and 3-NPA + Orientin ab. The biologically primary comparisons were Control versus 3-NPA and 3-NPA versus 3-NPA + Orientin. For both markers, the 3-NPA versus 3-NPA + Orientin comparison was not significant (adjusted *p* = 1.000).

With 8-OHdG staining, strong, coarse granular immunopositivity was observed in cortical pyramidal neurons and striatal neurons in the 3-NPA group, predominantly outside the nigrostriatal tract bundles (Figure 4G). IRS values differed among groups (Kruskal–Wallis H(3) = 19.43, *p* < 0.001; Figure 5, left panel). The Dunn–Bonferroni-adjusted pairwise *p* values were as follows: Control versus Orientin, *p* = 1.000; Control versus 3-NPA, *p* = 0.0015; Control versus 3-NPA + Orientin, *p* = 0.0482; Orientin versus 3-NPA, *p* = 0.0039; Orientin versus 3-NPA + Orientin, *p* = 0.0989; and 3-NPA versus 3-NPA + Orientin, *p* = 1.000. Thus, staining appeared lower in the combined-treatment images, but the IRS comparison with 3-NPA alone was not significant.

In the 3-NPA group, 4-HNE immunoreactivity was more prominent in myelinated nigrostriatal tract bundles than in striatal neuronal cell bodies (Figure 4K). IRS values differed among groups (Kruskal–Wallis H(3) = 18.87, *p* < 0.001; Figure 5, right panel). The Dunn–Bonferroni-adjusted pairwise *p* values were as follows: Control versus Orientin, *p* = 1.000; Control versus 3-NPA, *p* = 0.0008; Control versus 3-NPA + Orientin, *p* = 0.0694; Orientin versus 3-NPA, *p* = 0.0062; Orientin versus 3-NPA + Orientin, *p* = 0.2875; and 3-NPA versus 3-NPA + Orientin, *p* = 1.000. Thus, staining appeared lower in the combined-treatment images, but the IRS comparison with 3-NPA alone was not significant. GFAP-positive astrocyte distribution, process morphology, and immunoreactivity did not differ among groups (Figure 4M–P).

## 4. Discussion

The principal finding was an endpoint-dependent response rather than uniform protection. Relative to 3-NPA alone, orientin co-administration increased GSH, SOD, and CAT and reduced MDA, plasma caspase-3, and plasma MCP-1. In contrast, GPx, COX-2, and iNOS did not differ significantly between the 3-NPA and combined-treatment groups, and GFAP did not differ among groups. Histology suggested less extensive striatal injury with co-treatment. The 8-OHdG and 4-HNE images showed a directionally lower staining pattern, but their 3-NPA + Orientin versus 3-NPA IRS comparisons were not significant. These results support a partial biochemical and tissue-level profile and do not establish a uniform neuroprotective effect or a mechanism involving Nrf2, NF-κB, or MCP-1 signaling.

The reductions in GSH, SOD, GPx, and CAT and the increase in MDA after 3-NPA are consistent with oxidative stress following complex II inhibition. Kim et al. [15] also reported an association between oxidative stress and inflammatory-protein expression in a 3-NPA model of striatal toxicity. In the present study, SOD and CAT values in the combined-treatment group did not differ from control values, whereas GSH remained lower than in both non-3-NPA groups. GPx did not differ between the 3-NPA and combined-treatment groups. This pattern indicates that the measured antioxidant endpoints did not respond uniformly and should not be interpreted as evidence of global restoration of antioxidant defenses.

Orientin has been associated with antioxidant responses in other neurotoxicity models. In a rotenone model and SH-SY5Y cells, orientin altered antioxidant markers and signaling readouts involving Nrf2-ARE, PI3K/Akt, JNK-ERK1/2, and TLR4/NF-κB [8]. Orientin also reduced ROS, 4-HNE, and 8-OHdG and altered Nrf2/HO-1 readouts in Aβ1-42-treated mice [16] and orientin-associated effects have been reported in cerebral ischemia [17]. These studies provide biological context but do not demonstrate that the same pathways mediated the present findings, because those pathways were not measured here. In the current dataset, MDA was lower with co-treatment, whereas the 8-OHdG and 4-HNE IRS comparisons between 3-NPA and combined treatment were not significant. Accordingly, the IHC results indicate a descriptive staining trend rather than confirmed normalization of oxidative damage.

Plasma caspase-3 was higher after 3-NPA and lower in the combined-treatment group. This endpoint is compatible with altered apoptotic activity but does not by itself establish suppression of the apoptotic cascade or identify an upstream pathway. Prior cell studies found that orientin reduced caspase-3-related readouts after oxygen–glucose deprivation/reperfusion or H_2_O_2_ exposure [18,19]. Those mechanistic findings should not be transferred to the present in vivo model without direct pathway measurements.

Among the measured inflammatory endpoints, MCP-1 differed between the 3-NPA and combined-treatment groups, whereas COX-2 and iNOS did not. MCP-1 also remained higher in the combined-treatment group than in the Orientin-only group. Studies in spinal-cord injury and cerebral ischemia models have reported orientin-associated changes in p38MAPK/iNOS or TLR4/NF-κB/TNF-α signaling [20,21]. A separate hypoxia model reported changes in MDA, iNOS, and caspase-3 after orientin treatment [22]. Because models, tissues, doses, and endpoints differ, these reports provide context only. The present data show an MCP-1 concentration difference but do not demonstrate MCP-1-dependent causality, monocyte recruitment, or suppression of a defined inflammatory pathway.

Histopathological examination showed striatal neuronal degeneration, neuronophagia, and gliosis in the 3-NPA group. These lesions appeared less extensive in representative combined-treatment sections, although residual necrotic and chromatolytic neurons remained. Classical 3-NPA studies describe neuronal/glial loss in the lesion core and astroglial responses in surrounding tissue [23], and a recent study linked 3-NPA injury to NLRP3, microglial, and astrocyte changes [24]. GFAP did not differ among groups in the present study. Therefore, the descriptive H&E pattern should be interpreted separately from the non-significant GFAP result and should not be used to infer a specific glial mechanism.

The spatial distributions of 8-OHdG and 4-HNE differed: 8-OHdG was most apparent in neuronal nuclear and cytoplasmic regions, whereas 4-HNE was concentrated in myelinated nigrostriatal bundles. This observation is descriptive. Although representative combined-treatment images showed less staining, the corresponding IRS comparisons with the 3-NPA group were not significant for either marker. Thus, the IHC findings do not provide statistical evidence that orientin reduced these markers under the present conditions.

The incremental contribution of this study is therefore narrow. Orientin’s antioxidant and anti-inflammatory actions have been reported in several in vivo models [16,17,20,21,22,25,26,27]. The present work adds a short-term 3-NPA dataset that jointly reports responsive and nonresponsive biochemical endpoints together with striatal morphology. It does not establish functional benefit, superior efficacy, or a new mechanism. Future experiments should combine behavior, region-specific biochemical sampling, synaptic or neuronal-loss measures, and prespecified pathway or target-intervention experiments.

The principal limitations are the small sample size (*n* = 6) and use of female animals only, a single dose and time point, hemispheric brain homogenates without regional microdissection for oxidative-stress assays, and plasma rather than brain measurements for caspase-3 and inflammatory markers. Histopathological injury was described morphologically, and no behavioral or other functional readout was obtained. The study also lacked direct Nrf2/NF-κB measurements, MCP-1 neutralization or another causal intervention, pharmacokinetic assessment, and blood–brain barrier measurements. These limitations preclude claims of functional neuroprotection or a specific molecular mechanism.

## 5. Conclusions

In this seven-day 3-NPA mouse model, orientin co-administration was associated with higher GSH, SOD, and CAT; lower MDA, plasma caspase-3, and plasma MCP-1; and less extensive injury in representative striatal sections than 3-NPA alone. The response was partial: GPx, COX-2, and iNOS did not differ between the 3-NPA and combined-treatment groups, MCP-1 remained higher than in the Orientin-only group, 8-OHdG and 4-HNE IRS comparisons between 3-NPA and combined treatment were not significant, and GFAP did not differ among groups. These findings describe an endpoint-dependent biochemical and histopathological profile. Without behavioral testing or direct pathway and target-intervention experiments, they do not demonstrate functional neuroprotection or define a molecular mechanism.

## Figures and Tables

**Table 1:** Descriptive statistics for each experimental group (mean ± SD; *n* = 6/group).

Endpoint	Control	Orientin	3-NPA	3-NPA + Orientin
GSH (mmol/g)	1.102 ± 0.155	1.018 ± 0.160	0.423 ± 0.098	0.710 ± 0.138
MDA (nmol/g)	3.999 ± 0.548	3.627 ± 0.373	8.739 ± 1.228	4.882 ± 0.682
SOD (U/g protein)	0.525 ± 0.075	0.517 ± 0.061	0.312 ± 0.055	0.452 ± 0.056
GPx (U/g)	0.150 ± 0.020	0.155 ± 0.051	0.067 ± 0.012	0.117 ± 0.016
CAT (kat/g)	0.000165 ± 0.000036	0.000164 ± 0.000037	0.000057 ± 0.000021	0.000146 ± 0.000026
Caspase-3 (pmol/L)	2.698 ± 0.172	2.544 ± 0.177	3.624 ± 0.256	2.882 ± 0.240
COX-2 (ng/mL)	1.6277 ± 0.0024	1.6274 ± 0.0026	1.6342 ± 0.0035	1.6297 ± 0.0042
iNOS (ng/mL)	1.595 ± 0.188	1.500 ± 0.104	1.875 ± 0.164	1.725 ± 0.111
MCP-1 (pg/mL)	62.77 ± 6.47	55.65 ± 3.05	80.22 ± 7.66	69.56 ± 5.19

Note. Brain-tissue endpoints are GSH, MDA, SOD, GPx, and CAT; plasma endpoints are caspase-3, COX-2, iNOS, and MCP-1.

**Table 2:** Two-way ANOVA main effects and interaction effects for parametric endpoints.

Endpoint	Effect	df	F	Exact *p*	ηp^2^
GSH	3-NPA	1, 20	74.834	3.41 × 10^−8^	0.789
GSH	Orientin	1, 20	3.191	0.0892	0.138
GSH	3-NPA × Orientin	1, 20	10.547	0.0040	0.345
MDA	3-NPA	1, 20	89.394	8.05 × 10^−9^	0.817
MDA	Orientin	1, 20	44.460	1.72 × 10^−6^	0.690
MDA	3-NPA × Orientin	1, 20	30.187	2.23 × 10^−5^	0.601
SOD	3-NPA	1, 20	29.756	2.44 × 10^−5^	0.598
SOD	Orientin	1, 20	6.697	0.0176	0.251
SOD	3-NPA × Orientin	1, 20	8.411	0.0089	0.296
CAT	3-NPA	1, 20	25.309	6.40 × 10^−5^	0.559
CAT	Orientin	1, 20	12.042	0.0024	0.376
CAT	3-NPA × Orientin	1, 20	12.694	0.0019	0.388
Caspase-3	3-NPA	1, 20	52.068	5.52 × 10^−7^	0.722
Caspase-3	Orientin	1, 20	26.129	5.32 × 10^−5^	0.566
Caspase-3	3-NPA × Orientin	1, 20	11.286	0.0031	0.361
COX-2	3-NPA	1, 20	10.970	0.0035	0.354
COX-2	Orientin	1, 20	3.149	0.0912	0.136
COX-2	3-NPA × Orientin	1, 20	2.566	0.1249	0.114
iNOS	3-NPA	1, 20	17.930	0.0004	0.473
iNOS	Orientin	1, 20	4.217	0.0533	0.174
iNOS	3-NPA × Orientin	1, 20	0.214	0.6486	0.011
MCP-1	3-NPA	1, 20	43.125	2.13 × 10^−6^	0.683
MCP-1	Orientin	1, 20	13.855	0.0013	0.409
MCP-1	3-NPA × Orientin	1, 20	0.547	0.4681	0.027

Note. Exact *p* values were calculated from the supplied F statistics with df1 = 1 and df2 = 20. Partial eta-squared was calculated from F and the residual degrees of freedom. GPx is excluded because it was analyzed using Kruskal–Wallis/Dunn–Bonferroni.

## Data Availability

The data presented in this study are available on request from the corresponding author. The data are not publicly available due to ethical restrictions regarding raw animal experimental data.

## Supplementary Materials

The following supporting information can be downloaded at: https://www.mdpi.com/article/10.3390/biology15191776/s1, Table S1: Hemispheric brain oxidative-stress endpoints; Table S2: Plasma apoptotic and inflammatory endpoints; Table S3: Immunohistochemical IRS summaries; Table S4: Dunn-Bonferroni adjusted IRS comparisons.

## Abbreviations

3-NPA: 3-nitropropionic acid; 4-HNE: 4-hydroxynonenal; 8-OHdG: 8-hydroxy-2′-deoxyguanosine; ARE: Antioxidant response element; CAT: Catalase; COX-2: Cyclooxygenase-2; GFAP: Glial fibrillary acidic protein; GPx: Glutathione peroxidase; GSH: Glutathione; H&E: Hematoxylin and eosin; iNOS: Inducible nitric oxide synthase; IRS: Immunoreactive score; MCP-1: Monocyte chemoattractant protein-1; MDA: Malondialdehyde; NF-κB: Nuclear factor kappa-light-chain-enhancer of activated B cells; Nrf2: Nuclear factor erythroid 2-related factor 2; ROS: Reactive oxygen species; SOD: Superoxide dismutase.
