# Supplementary material for: Endpoint-Dependent Biochemical and Histopathological Effects of Orientin in a Short-Term 3-Nitropropionic Acid Mouse Model
Source: Biology (Basel). 2026 Oct 7;15(19):1776. doi: 10.3390/biology15191776 (PMC13664224; doi:10.3390/biology15191776)
Supplement: Supplementary file 1 [file biology-15-01776-s001.zip › biology-4609112-supplementary.pdf]

Supplementary Statistical Tables

Two-way factorial analyses

Analysis. The original animal-level workbook was analyzed as a balanced 2 × 2 design with 3-NPA exposure and orientin treatment as fixed factors (n = 6 per cell). Each table reports sums of squares (SS), degrees of freedom (df), mean squares (MS), F statistics, and two-sided p values. GPx was not included because it did not meet the manuscript's parametric assumptions and remained under the prespecified Kruskal-Wallis/Dunn-Bonferroni analysis.

Table S1 Hemispheric brain oxidative-stress endpoints

Endpoint	Effect	SS	df	MS	F	p
GSH	3-NPA	1.4620218	1	1.4620218	74.834	<0.001
GSH	Orientin	0.062350988	1	0.062350988	3.191	0.089
GSH	3-NPA × Orientin	0.20606047	1	0.20606047	10.547	0.004
GSH	Residual	0.39073781	20	0.019536891		
MDA	3-NPA	53.9238	1	53.9238	89.394	<0.001
MDA	Orientin	26.819361	1	26.819361	44.460	<0.001
MDA	3-NPA × Orientin	18.209459	1	18.209459	30.187	<0.001
MDA	Residual	12.064355	20	0.60321777		
SOD	3-NPA	0.11501964	1	0.11501964	29.756	<0.001
SOD	Orientin	0.025887586	1	0.025887586	6.697	0.018
SOD	3-NPA × Orientin	0.032510904	1	0.032510904	8.411	0.009
SOD	Residual	0.077309505	20	0.0038654752		
CAT	3-NPA	2.4289749e-08	1	2.4289749e-08	25.309	<0.001
CAT	Orientin	1.1556812e-08	1	1.1556812e-08	12.042	0.002
CAT	3-NPA × Orientin	1.2182254e-08	1	1.2182254e-08	12.694	0.002
CAT	Residual	1.9194445e-08	20	9.5972223e-10		

Note. SS, sum of squares; MS, mean square. Residual df = 20 for each endpoint. Values are calculated from the original animal-level measurements.

Table S2 Plasma apoptotic and inflammatory endpoints

Endpoint	Effect	SS	df	MS	F	p
Caspase-3	3-NPA	2.3951646	1	2.3951646	52.068	<0.001
Caspase-3	Orientin	1.2019492	1	1.2019492	26.129	<0.001
Caspase-3	3-NPA × Orientin	0.51914093	1	0.51914093	11.286	0.003
Caspase-3	Residual	0.92000548	20	0.046000274		
COX-2	3-NPA	0.0001159186	1	0.0001159186	10.970	0.003
COX-2	Orientin	3.3277498e-05	1	3.3277498e-05	3.149	0.091
COX-2	3-NPA × Orientin	2.7118201e-05	1	2.7118201e-05	2.566	0.125
COX-2	Residual	0.00021133424	20	1.0566712e-05		
iNOS	3-NPA	0.38260903	1	0.38260903	17.930	<0.001
iNOS	Orientin	0.089977849	1	0.089977849	4.217	0.053
iNOS	3-NPA × Orientin	0.0045677024	1	0.0045677024	0.214	0.649
iNOS	Residual	0.42677581	20	0.02133879		
MCP-1	3-NPA	1475.0535	1	1475.0535	43.125	<0.001
MCP-1	Orientin	473.8836	1	473.8836	13.855	0.001
MCP-1	3-NPA × Orientin	18.705175	1	18.705175	0.547	0.468
MCP-1	Residual	684.0812	20	34.20406		

Note. SS, sum of squares; MS, mean square. Residual df = 20 for each endpoint. Values are calculated from the original animal-level measurements.

Table S3 Immunohistochemical IRS summaries

Marker	Group	n	Median	Q1	Q3
8-OHdG	Control	6	1	1	2
8-OHdG	Orientin	6	1.5	1	2
8-OHdG	3-NPA	6	6	6	9
8-OHdG	3-NPA + Orientin	6	5	4	6
4-HNE	Control	6	1	1	2
4-HNE	Orientin	6	2	1	2
4-HNE	3-NPA	6	6	4	6
4-HNE	3-NPA + Orientin	6	4	3	4

Note. IRS range, 0-9. Quartiles use the median of the lower and upper three observations.

Table S4 Dunn-Bonferroni adjusted IRS comparisons

Marker	Group 1	Group 2	Adjusted p
8-OHdG	Control	Orientin	1.000
8-OHdG	Control	3-NPA	0.0015
8-OHdG	Control	3-NPA + Orientin	0.0482
8-OHdG	Orientin	3-NPA	0.0039
8-OHdG	Orientin	3-NPA + Orientin	0.0989
8-OHdG	3-NPA	3-NPA + Orientin	1.000
4-HNE	Control	Orientin	1.000
4-HNE	Control	3-NPA	0.0008
4-HNE	Control	3-NPA + Orientin	0.0694
4-HNE	Orientin	3-NPA	0.0062
4-HNE	Orientin	3-NPA + Orientin	0.2875
4-HNE	3-NPA	3-NPA + Orientin	1.000

Note. 8-OHdG: $H(3) = 19.43$, $p < 0.001$. 4-HNE: $H(3) = 18.87$, $p < 0.001$. Six pairwise comparisons were Bonferroni adjusted for each marker.
